# Performance-based metacognitive tests versus self-report: what does prediction tell us?

**DOI:** 10.1186/s41155-025-00337-2

**Published:** 2025-10-04

**Authors:** Jhonys de Araujo, Cristiano Mauro Assis Gomes, Enio Galinkin Jelihovschi

**Affiliations:** 1 Laboratory for Cognitive Architecture Mapping (LAICO), Department of Psychology, Universidade Federal de Minas Gerais (UFMG), Campus Pampulha, Av. Pres. Antônio Carlos, 6627 Pampulha, Belo Horizonte, MG CEP: 31270-901 Brazil; 2 Department of Exact and Technological Sciences, Universidade Estadual de Santa Cruz (UESC), Campus Soane Nazaré de Andrade, Nazaré de Andrade Rodovia Ilhéus-Itabuna km, Ilhéus, BA CEP: 16.45.662-900 Brazil

**Keywords:** Academic achievement, Performance-based tests, Metacognition, Prediction, Regulation of cognition, Structural equation modeling

## Abstract

**Background:**

The measurements of metacognition through performance-based tasks are better predictors of academic performance than those based on self-report tests, but evidence on the prediction of academic performance by standardized performance-based metacognition tests is scarce. The reason is that there are few tests of this nature with psychometric evidence of validity and reliability. Only a single study with Honduran university students compared the prediction of academic performance by a standardized performance-based test, and a self-report test in which both measure cognition regulation, a metacognitive construct. The results indicated that only the standardized performance-based test predicts academic performance, and the measures of these tests are not correlated.

**Objective:**

Two hypotheses are investigated in this article: (1) performance-based metacognitive tests predict academic performance better than self-report metacognitive tests; (2) there is a null correlation between measures of cognition regulation from performance-based standardized tests and self-report tests.

**Method:**

A sample of 264 university students and graduates from Brazil, with an average age of 21.1 years, is used in the study. The majority are female, from private institutions, and enrolled in humanities and social sciences courses. The Meta-Text was used as the standardized performance-based test, and the self-report test was the Metacognitive Self-Regulation Scale of the Motivated Strategies for Learning Questionnaire (MSLQ). The predictors were cognition regulation, measured by both tests, and judgment, measured by the Meta-Text. The outcome was the overall score on the National High School Exam, a large-scale educational assessment for university admission.

**Results:**

Only the regulation of cognition measured by Meta-Text predicts academic performance (*β* = 0.47, CI 95% [0.36, 0.58]). The correlations between the test measures were null (*r* = .002, *p* = .974).

**Conclusion:**

The evidence corroborated both hypotheses and raises doubts about the quality of self-report tests for measuring cognition regulation. It also indicates that standardized performance-based tests have a similar predictive capacity to tasks that require performance. This result is promising because standardized tests are easy to apply and correct, allowing studies to be carried out on large samples, while performance-based tasks require a complex process, only feasible in studies on small samples.

## Introduction

Metacognition is defined as cognition that operates on cognition itself (Flavell, 1979). The field of metacognition studies has several distinct models; however, there is a consensus that metacognition encompasses two broad domains: metacognitive knowledge and cognitive regulation. The former refers to the knowledge individuals have about themselves and their cognitive processes, and the latter pertains to the action of processes that regulate cognition itself (Stephanou & Mpiontini, 2017). There is also consensus that metacognition is strongly associated with academic performance. According to the arguments of Craig et al., (2020, p.156): “Metacognition, then, is essential for learning, and training metacognitive skills has been repeatedly shown to increase academic achievement.” Therefore, predictive evidence of metacognition on academic performance is very important for evaluating the quality of metacognition measurement. Not surprisingly, academic performance is frequently used in predictive studies on metacognition (Dent & Koenka, 2016; Ohtani & Hisasaka, 2018; Richardson et al., 2012).

The use of self-report tests and performance-based tasks to measure metacognition is common (Ozturk, 2017). Self-report tests are standardized measurement instruments that typically require respondents to evaluate statements reflecting behaviors indicative of the construct being assessed. In these tests, respondents often rate the extent to which these behaviors are part of their lives. This evaluation is then used to measure the construct. In contrast, performance-based measures require respondents to perform specific behaviors, and their performance is used to assess the construct. In summary, self-report tests rely on respondents’ evaluations as data for measurement, whereas performance-based measures use performance data for the same purpose (American Educational Research Association [AERA], American Psychological Association [APA], & National Council on Measurement in Education [NCME], 2014).

The field of studies on metacognition presents strong evidence that the measure of metacognition by tasks that require performance is better at predicting academic performance than the measure from self-report tests. The meta-analysis by Ohtani and Hisasaka (2018) found a correlation of 0.53, 95% CI [0.45, 0.61] between academic performance and the measure of metacognition by tasks requiring performance. This correlation drops to 0.19, 95% CI [0.16, 0.23] when the measure of metacognition comes from self-report tests. In predictive terms, these results indicate that metacognition measured by performance-based tasks predicts 28.09% of academic performance, while the measurement of metacognition by self-report-based tests predicts only 3.61% of academic performance. The meta-analyses of Dent and Koenka (2016) and Richardson et al. (2012) reinforce the results of Ohtani and Hisasaka (2018). Dent and Koenka (2016) reported a correlation of 0.39, 95% CI [0.34, 0.43] between academic performance and the measure of metacognition for tasks requiring performance. On the other hand, Richardson et al. (2012) reported a correlation of 0.18, 95% CI [0.10, 0.26] between academic performance and metacognition measured by self-report tests.

Even though this evidence indicates better prediction of academic performance by measures that require performance in relation to self-report, it does not address prediction from standardized performance-based tests. The field of metacognition still lacks standardized performance-based tests to measure its constructs. We know of only two standardized performance-based metacognition tests that have psychometric evidence of validity and reliability. These are the Metacognitive Monitoring Test (Castillo Diaz & Gomes, 2022; Gomes & Golino, 2014; Gomes et al., 2014, 2021) and the Meta-Text (Castillo-Diaz & Gomes, 2021, 2023; Castillo-Diaz et al., 2022). This shows how little evidence there is on the prediction of academic performance by metacognition as measured by standardized performance-based tests.

The studies by Gomes et al. (2014) and Castillo-Diaz and Gomes (2022, 2023) provide initial and promising evidence on this issue. Gomes et al. (2014) found that metacognitive monitoring, measured by the Metacognitive Monitoring Test, predicted 20.43% of the variance in the overall school performance factor, estimated from students’ grades in Mathematics, Portuguese, Geography, and History, with a sample consisting of primary and secondary school students in Brazil. Castillo-Diaz and Gomes (2022) found that metacognitive monitoring measured by the Metacognitive Monitoring Test predicted 40.96% of academic performance measured by a large-scale educational test used for university admission in Honduras, with a sample made up of university students from a large Honduran public university. Subsequently, Castillo-Diaz and Gomes (2023) applied the Meta-Text at this same Honduran university and included a metacognitive test based on self-report, the Metacognitive Awareness Inventory (MAI) (Schraw & Dennison, 1994). Both tests measure the broad metacognitive domain of cognition regulation. They found that only cognition regulation measured by the Meta-Text was able to predict academic performance. Cognition regulation predicted 14.44% of academic performance as measured by the overall university grade, as well as predicting 65.61% of performance as measured by a large-scale educational assessment. In this study, the measures from the MAI did not show any predictive role with regard to academic performance. In addition, its measures showed zero correlation with the Meta-Text measures. This result is surprising, given that both the MAI and Meta-Text measure the broad metacognitive domain of cognitive regulation. The zero correlation between these measures indicates the possibility that the MAI or Meta-Text is not actually measuring the construct it purports to measure. Considering that cognition regulation is a domain that involves not metacognitive knowledge, but the action of metacognition on cognitive tasks, it is likely that Meta-Text is more appropriate for measuring this construct. In addition, its prediction of academic performance is much higher, which corroborates this argument. Although still at the beginning, this evidence suggests that standardized performance-based metacognition tests are much better at identifying the predictive power of metacognition on academic performance than standardized self-report tests.

The study by Castillo-Diaz and Gomes (2023) was an initial milestone in studies comparing the predictive power of performance-based metacognitive tests compared to self-report metacognitive tests. Although its evidence is promising, a single study is not enough to generalize. New studies are needed in different contexts. In this article, we take a further step in this research agenda. As in the study by Castillo-Diaz and Gomes (2023), we used Meta-Text for the performance-based measure of the broad domain of cognition regulation and the specific metacognitive component of judgment. We used the Metacognitive Self-Regulation Scale of the Motivated Strategies for Learning Questionnaire (MSLQ) for the self-report-based measure of cognition regulation (Pintrich et al., 1991), since the MSLQ is a test of recognized importance in the area of studies on metacognition and self-regulated learning (Duncan & McKeachie, 2005).

Two hypotheses will be evaluated in this article. The first assumes that performance-based metacognitive tests have greater predictive ability than self-report metacognitive tests for predicting academic performance. The second assumes that there is a null correlation between measures of the broad metacognitive domain of cognition regulation from performance-based tests and self-report-based tests.

To investigate these hypotheses, we used structural equation modeling to analyze three predictive models. Partial model 1 analyzes the prediction of academic performance by the self-report test (MSLQ Metacognitive Self-Regulation Scale). Partial model 2 investigates the prediction of academic performance by the performance-based test (Meta-Text). The complete model integrates the partial models. This model makes it possible to compare the predictive power of both tests for predicting academic performance and to analyze the correlations of the Meta-Text measures with the MSLQ Metacognitive Self-Regulation Scale measure.

## Method

### Participants

The participants (*n* = 264) are mostly female (*n* = 186, 70.45%) and have an average age of 21.1 years (SD = 4.0). Almost all of them are higher education students from various Brazilian institutions (*n* = 245, 92.80%), while the rest have completed higher education (*n* = 19, 7.20%). The majority came from private institutions (*n* = 218, 82.60%) and from courses in the humanities and social sciences (*n* = 166, 62.89%); the remainder came from courses in health (*n* = 78, 29.54%), exact sciences (*n* = 18, 6.82%), and economic and administrative sciences (*n* = 2, 0.75%).

This sample size allows the models tested in this study to have a statistical power of 0.80 and an alpha significance level of 0.05 for effect sizes of at least 0.21. This information was calculated using the A-priori Sample Size Calculator for Structural Equation Models (Soper, 2024).

### Instruments

#### Meta-text

Meta-Text is a standardized performance-based test created by Castillo-Diaz and Gomes (2021) with the original aim of assessing the broad metacognitive domain of cognition regulation and its specific metacognitive abilities: planning, monitoring, and judgment. The abilities assessed by the test are defined as follows: planning is the ability to establish or select a sequence of steps or strategies for carrying out tasks; monitoring is the ability to identify errors during the execution of tasks; judgment is the individual’s assessment of their performance in tasks carried out (Castillo-Diaz & Gomes, 2021).

The test consists of 18 questions. Each question shows the following: (1) an author’s specific objective when trying to write a certain text; (2) five available sentences that could or could not have been used by the author to correctly achieve this objective; (3) a text produced by the author using some of these available sentences. In addition to these elements, there are three commands. Command A asks the respondent to analyze the steps needed to produce a correct text that meets the author’s objective. In this context, the respondents must select the sentences they would use to produce a correct text. Command A assesses the ability of planning. Command B asks the respondent to assess whether their answer to command A was right or wrong. Command B assesses the ability of judgment. Command C asks the respondent to assess whether the text produced by the author has achieved its objective; if not, the respondent is asked to identify the sentences selected incorrectly by the author and to identify which ones he failed to insert that were correct (Castillo-Diaz & Gomes,^ 2021^).

Each of the 18 questions presents the three commands previously described, and each command is a test item, totaling 54 items with different levels of difficulty. The planning and monitoring scores are coded as 1 when the respondent gets it right and 0 when they get it wrong, while the judgment score is coded as 1 when the respondent thinks they got the planning item right and 0 when they think they got it wrong. The 18 questions in the test are testlets, as the judgment, planning, and monitoring items are always linked to the same question. This characteristic of the test makes the modeling of the measure a little more complex, requiring the monitoring and planning items of each question to be correlated (Castillo-Diaz & Gomes, 2021).

Meta-Text has evidence of content validity (Castillo-Diaz & Gomes, 2021) and structural and predictive validity (Castillo-Diaz & Gomes, 2023; Castillo-Diaz et al., 2022), with a reliable measure for the latent variable of the broad metacognitive domain of cognition regulation and the latent variable judgment. Castillo-Diaz et al. (2022) tested several measurement models via confirmatory factor analysis and found that the model that best represented the Meta-Text measure was a bifactor model with a general latent variable, the domain of cognition regulation, and a specific latent variable, judgment, orthogonalized to each other (*χ*^2^ [1341] = 1727.78, CFI (Confirmatory Fit Index) = 0.992; RMSEA (Root Mean Square Error of Approximation) = 0.021, 90% CI [0.018, 0.024]). These latent variables exhibited a McDonald’s omega equal to or greater than 0.70.

#### Metacognitive self-regulation scale

The Metacognitive Self-Regulation Scale is one of the scales of the MSLQ, an instrument developed by Pintrich et al. (1991) to measure self-regulated learning in a wide variety of components. The MSLQ is underpinned by the sociocognitive perspective, which recognizes that students can regulate their own learning process through the management of learning strategies and motivations and uses various theoretical elements to underpin its assessed motivational and strategic components, such as self-efficacy theory, expectancy-value theory, and metacognition (Duncan & McKeachie, 2005). It is widely used to assess self-regulatory components in higher education (Roth et al., 2016) and has been studied in various countries and cultural contexts (Duncan & McKeachie, 2005). The instrument includes 15 scales that measure different self-regulatory components, six of which are motivational and nine strategic, and consists of 81 items, each containing a sentence that describes a self-regulatory behavior that the student can manifest in the academic context. Each item is answered using a seven-point Likert scale: the lowest value indicates that the behavior is not very representative, while the highest value indicates that it is very representative of the student's self-regulatory behavior.

The Metacognitive Self-Regulation Scale assesses the student's metacognition, more specifically their regulation of cognition. It is one of the nine scales that assess the strategic learning components evaluated by the MSLQ. It consists of 12 items that express planning, monitoring, and regulating behaviors that occur in the academic sphere (Pintrich et al., 1991). In the theoretical conception of this scale, planning is the behavior of defining objectives and analyzing study material in a structured way, while monitoring refers to maintaining focus and understanding. The term regulation, on the other hand, is not conceived as a broad dimension, as in Meta-Text, but rather as a specific aspect of metacognition, responsible for adjusting strategies that allow learning to be corrected or optimized, such as changing the way the material is read when comprehension is difficult (Pintrich et al., 1991). Despite the difference in nomenclature, the metacognitive self-regulation component of the MSLQ is the cognition regulation domain of the Meta-Text; with different names, both deal with the same domain, that is, a broad component of metacognition that articulates and integrates the specific metacognitive components of online control of cognition over cognition itself, such as monitoring and planning.

Araujo et al. (2023) investigated the structural validity of the complete MSLQ, with all its 15 scales and 81 items. The authors tested different models, and they found that the most suitable model was the bifactorial one, consisting of a general self-regulated learning component, four broad components, and 15 specific components (*χ*^2^ [3069] = 15,021.14; CFI = 0.932; RMSEA = 0.076, 90% CI [0.075, 0.078]). The Metacognitive Self-Regulation Scale had a Cronbach’s alpha of 0.77 and a McDonald’s omega of 0.32. The authors reported that most of the items in this component had a factor loading of less than 0.30 in the specific metacognitive self-regulation component, but a significant loading in the general self-regulated learning component (Araujo et al., 2023). This could indicate that the items on the Metacognitive Self-Regulation Scale are better explained by the general component of self-regulated learning than by the specific component. However, this result comes from a single study, so further studies are needed to reach a better-established conclusion on this issue.

### Data collection

The participants in this study were selected for convenience. Participants were invited through personal and virtual contact with higher education institutions, professors, researchers, and students. Data were collected virtually in the first semester of 2023, after approval by the Ethics Committee (61833722.0.0000.5149). Participants received instructions on how to take part and a link directing them to an online data collection environment, Psytoolkit (Stoet, 2010, 2017). After agreeing to the conditions stated, the participants answered a sociodemographic questionnaire. In this questionnaire, they were also asked to state their overall score from the last time they took the National High School Exam (ENEM). The ENEM is a large-scale educational assessment widely used for admission to public universities in Brazil. It consists of 180 multiple-choice questions and a written essay, administered over two consecutive Sundays with durations of 5 and 4 h, respectively. Using item response theory, it provides measures for the domains of mathematics, natural sciences, humanities, languages, the essay, and an overall student score. For each of these measures, the ENEM provides a metric with a mean of 500 points and a standard deviation of 100 points (INEP, 2021). The participants then answered the Meta-Text and Metacognitive Self-Regulation, respectively. Participants were informed that their participation would take around 60 min, but there was no time limit on the platform. After taking part, participants received immediate feedback on their answers. This feedback provided a brief explanation of what had been assessed and showed the participant how their self-assessment and performance fared.

### Data analysis

The analyses were divided into two stages. In the first, three predictive models were tested using structural equation modeling: partial model 1, partial model 2, and the complete model. Each of these models has two parts: the measurement model, which estimates the relationships between the latent variables and their respective observable variables, and the structural relationship model, which, in the case of this study, estimates the predictive role of the latent variables measured in relation to the outcome variable, the overall ENEM score. The weighted least square mean and variance (WLSMV) estimator was used in all models, since the items from the MSLQ and Meta-Text are categorical.

Partial model 1 measures the latent variable of cognition regulation, which explains the variance of the 12 items of the MSLQ Metacognitive Self-Regulation Scale. There is a correlation between items 33 and 57 of the MSLQ because they are the only items on the Scale where their original score goes in the opposite direction to the scale measure. The original score of these two items was inverted, and this inversion was used in both the partial model 1 and the complete model. The structural relationship model of partial model 1 defines that cognition regulation predicts the overall ENEM score.

Partial model 2 has a bifactor structure as its measurement model. In this, the general latent variable cognition regulation explains the variance of all 54 items in the Meta-Text, while the latent variable judgment explains the variance of 18 items in the Meta-Text, all of which measure judgment. In the bifactor structure, the cognition regulation latent variable is orthogonalized to the judgment latent variable. The measurement model also includes 18 correlations between the planning items and the monitoring items of the same question. The structural relationship model of partial model 2 defines that the Meta-Text latent variables of judgment and cognition regulation predict the overall ENEM score.

The complete model is the integration of the two partial models, with all their relationships. The structural relations model of the complete model estimates the correlations between the cognition regulation of the MSLQ Metacognitive Self-Regulation Scale and the cognition regulation and judgment of the Meta-Text.

In bifactor models, in which there is a general latent variable and specific latent variables, all orthogonalized to each other, it is common for an item to have a negative loading on a latent variable, not because this loading is true, but because the variance of this item is only explained by another latent variable in the model. In this case, this loading is just a result of the orthogonalization of the general factor in relation to a specific factor. If this happened in the measurement models of partial model 2 and the complete model, the negative loadings would be constrained to zero.

Any tested model would be rejected if it had a CFI, TLI (Tucker Lewis Index), or GFI (Goodness-of-Fit Index) < 0.90 or RMSEA ≥ 0.10 (Thakkar, 2020). Regarding the reliability of the latent variables of the measurement model, we consider those with Cronbach’s alpha and McDonald's omega ≥ 0.60 to be reliable (Gomes et al., 2020; Valentini et al., 2015).

The second stage generated descriptive statistics on:

1. The overall ENEM score.
2. The percentage of the total raw score of the cognition regulation measure of the MSLQ Metacognitive Self-Regulation, which indicates the extent to which participants perceive themselves as using cognition regulation.
3. The percentage of the total raw score of the Meta-Text cognition regulation measure, indicating the participants’ performance in relation to cognition regulation.
4. The percentage of the total raw score of the Meta-Text judgment measure, indicating how well the participants judged their performance.

Only the items that had positive loading on the respective latent variables of the measurement models of the three predictive models tested were used to calculate the percentage of the total scores of the study’s predictor variables.

Not all participants answered all the items in all the tests. Only data from participants who answered at least 80% of the items that made up the two tests together and at least 50% of the items of any measure from one of the tests were included in the analyses.

The analyses were carried out in R v. 4.2.2 (R Core Team, 2022). The first stage used the lavaan v. 0.6–17 (Rosseel, 2023) and semTools v. 0.5–6 (Jorgensen, 2022) packages, while the second stage used the psych v. 4.2.3 (Revelle, 2024) package.

## Results and discussion

All the Meta-Text planning and monitoring items and all the MSLQ items were answered by the participants. Thirty-five participants failed to answer some items of the Meta-Text judgment. The average number of unanswered judgment items was 1.49 (SD = 0.92), ranging from 1 to 5 items. These unanswered items correspond to only 0.3% of the total responses to the Meta-Text and MSLQ items together, as well as only 1.1% of the total responses to the Meta-Text judgment items, representing a very small portion.

The descriptive statistics of the variables in this study are shown in Table 1. The distribution of the overall ENEM score is important because it is the outcome variable of the analyzed predictive models. The results indicate that the distribution of the data in this variable has two favorable properties: heterogeneity, i.e., it encompasses a wide range of performances, shown by the large difference between the minimum and maximum values, and univariate normal distribution, indicated by kurtosis and skewness.

**Table 1:** Descriptive statistics of the study variables

Variable	*M* (*SD*)	Minimum	Maximum	Kurtosis	Skewness
Overall ENEM Score (*n* = 264)	616.40 (84.83)	420	870	−0.24	0.22
Meta-Text Judgment (*n* = 229)	93.30% (13.42%)	0.00%	100.00%	14.65	−3.32
CR Meta-Text (*n* = 247)	37.06% (13.77%)	2.63%	71.05%	−0.75	0.06
CR MSLQ (*n* = 264)	66.43% (13.91%)	26.19%	98.81%	−0.34	−0.30

*Note. CR* broad metacognitive domain of cognition regulation, *M* mean, *SD* standard deviation

ENEM has a metric in which the average overall score is 500 points, with a standard deviation of 100 points (INEP, 2021). The average overall ENEM score of the participants in this study (*M* = 616.4) is more than one standard deviation above the average of this metric, suggesting that the average performance of the participants is high. However, this performance is still considerably below the ENEM cut-off scores for entry into many higher education courses at Brazilian public universities, many of which require a cut-off value of two, even three standard deviations above the mean for the candidate to be selected (MEC, 2023).

Table 1 also provides relevant information regarding the predictors of the predictive analyzed models. The average total raw judgment score for Meta-Text is very high (93.3%). The data for this variable shows a strong kurtosis of 14.65, indicating univariate non-normality. This is because the vast majority of participants judged that they got almost everything right. On the other hand, the average total raw score for regulating cognition on the Meta-Text was only 37.1%. In other words, on average, the participants rated themselves very highly, but on average, they scored poorly. This contrast means poor metacognitive accuracy.

The mean score of 66.4% on the MSLQ's Metacognitive Self-Regulation Scale (Table 1) indicates that participants perceive themselves as using the broad metacognitive domain of cognition regulation in a moderately superior way. This positive perception opposes the low performance in cognition regulation on the Meta-Text.

The raw total scores of the measures of cognition regulation on the MSLQ Metacognitive Self-Regulation Scale and the Meta-Text showed univariate normal distribution (kurtosis and skewness), as well as good heterogeneity, indicated by the maximum and minimum values. Despite this heterogeneity, there is a lack of very high raw total scores in the regulation of cognition on the Meta-Text (> 75%), probably because the test was more difficult than the level of comprehension of the students sampled. There is also a lack of very negative perceptions regarding the use of cognition regulation (< 25%) on the MSLQ, possibly because this test is self-reported and people tend to overestimate their ability.

Tables 2 and 3 summarize the most important information about the results of the measurement model and the structural relationship model of all three models tested. The most basic information in Tables 2 and 3 refers to the degree of fit of the models tested. All of them showed an acceptable fit, indicating that the results present in the models can be considered for the purposes of interpretation and discussion.

**Table 2:** Results of partial model 1 and partial model 2

Model	*χ*^2^(df)	CFI TLI GFI	RMSEA [CI 90%]	Measurement model	Structural relations model [CI 95%]
Partial model 1 MSLQ (*n* = 264)	126.73 (64)	.962 .954 .994	.061 [.045, .077]	**CR MSLQ:** Factor loadings (*M* = .48, *SD* = .19; MIN = .10, MAX = .67) Reliability *α* = .74 *ω* = .76 Correlation between items (*M* = .46, *SD* = .00; MIN = .46, MAX = .46)	CR MSLQ: *β* = 0.09 [− 0.04, 0.22] *R*^2^ = 0.01%
Partial model 2 Meta-Text (*n* = 264)	3884.28 (1410)	.904 .899 .916	.082 [.079, .085]	**CR Meta-Text:** Factor loadings (*M* = .34, *SD* = .30; MIN = .00, MAX = .81) Reliability *α* = .84 *ω* = .77 **Judgment Meta-Text:** Factor loadings (*M* = .81; *SD* = .08; MIN = .65; MAX = .93) Reliability *α* = .82 *ω* = .94 Correlation between items (*M* = .80; *SD* = .26 MIN = .00; MAX = 1.07)	CR Meta-Text: *β* = 0.47 [0.36, 0.58] Judgment Meta-Text: *β* = 0.02 [− 0.15, 0.19] *R*^2^ = 22.30%

*Note. χ*^*2*^*(gl)* chi-square and degrees of freedom, *CI* confidence interval, *CR* broad metacognitive domain of cognition regulation, *β* standardized beta of the predictor on the outcome, *R*^*2*^ coefficient of determination, which explains the variance of the outcome variable by the predictive model, *M* mean, *SD* standard deviation, *MIN* minimum, *MAX* maximum

Some items of partial model 2 and complete model could have the constraints of zero factor loading in the latent variable cognition regulation or judgment. This occurred in planning items 6 and 12, monitoring item 16, and judgment items 1, 3 to 9, 13 to 16, and 18, specifically in relation to the latent variable of regulating Meta-Text cognition.

**Table 3:** Results of complete model

Model	*χ*^2^(df)	CFI TLI GFI	RMSEA [CI 90%]	Measurement model	Structural relations model [CI 95%]
Complete Meta-Text and MSLQ (*n* = 264)	4876.20 (2120)	.900 .896 .910	.070 [.068, .073]	**CR MSLQ:** Factor loadings (*M* = .48, *SD* = .19; MIN = .11, MAX = .67) Reliability *α* = .74 *ω* = .76 **CR Meta-Text:** Factor loadings (*M* = .34, *SD* = .30; MIN = .00, MAX = .81) Reliability *α* = .82 *ω* = .77 **Judgment Meta-Text:** Factor loadings (*M* = .81, *SD* = .08; MIN = .65, MAX = .93) Reliability *α* = .89 *ω* = .94 Correlation between items (*M* = .80, *SD* = .26; MIN = .00, MAX = 1.07) Correlation between factors *r* = .002 (p = .974) CR Meta-Text and CR MSLQ *r* = − .007 (*p* = .941) CR MSLQ and judgment Meta-Text	CR Meta-Text: *β* = 0.47 [0.36, 0.58] Judgment Meta-Text: *β* = 0.02 [− 0.16, 0.20] CR MSLQ: *β* = 0.09 [− .33, 0.21] *R*^2^ = 23.10%

*Note. χ*^*2*^*(gl)* chi-square and degrees of freedom, *CI* confidence interval, *CR* broad metacognitive domain of cognition regulation, *β* standardized beta of the predictor on the outcome,* r* correlation, *R*^*2*^ coefficient of determination, which explains the variance of the outcome variable by the predictive model, *M* mean, *SD* standard deviation, *MIN* minimum, *MAX* maximum

The measurement model of each of the three models tested and shown in Tables 2 and 3 indicates that the estimated latent variables of the predictors had satisfactory average factor loadings, with a value equal to or greater than 0.34, as well as acceptable reliability, with alphas and omegas equal to or greater than 0.74. These results indicate that the measurement of the predictors shows evidence of validity and reliability.

The structural relationship model for each of the three models tested (Tables 2 and 3) provides important information on the predictive role of the study's predictors in relation to the outcome, i.e., the participants’ overall ENEM score. Partial model 1 only includes cognition regulation as measured by the MSLQ as a predictor. It is important to note that it has no predictive role in relation to the overall ENEM score of the participants in this study. The standardized beta of 0.09 is very low and not statistically significant, and the 95% confidence interval is between −0.04 and 0.22. The Metacognitive Self-Regulation Scale had a correlation of 0.30 with academic achievement in Pintrich et al. (1993). This correlation was obtained using the final grade assigned by instructors as the measure of academic performance. In our study, we used the score from a large-scale educational assessment as the measure of academic performance. It is possible that the difference in prediction found between our study and Pintrich et al. (1993) was due to the method of measuring academic performance.

An important issue addressed in this article is whether the prediction of metacognition on academic performance is well identified by performance-based tests. Our hypothesis is that performance-based tests are of higher quality compared to self-report tests since they have a high predictive power of metacognition on academic performance. The results presented in Tables 2 and 3 are quite clearly in favor of this hypothesis. The regulation of cognition measured by Meta-Text (partial model 2 and the complete model) is the only predictor that explains the variance in the overall ENEM score of the participants in the study. It is the only one with a good standardized beta in relation to the outcome as well as statistical significance. In the complete model (Table 3), cognition regulation measured by Meta-Text had a standardized beta of 0.47 on the outcome. This indicates that this predictor explains 22.1% of the variance (squaring the value and multiplying the result by 100) of the overall ENEM score of the study participants, which is a very satisfactory explanation for studies in the humanities and education. Taking the 95% confidence interval values of this beta [0.36, 0.58] as a reference, it can be said that, in the worst-case scenario, cognition regulation measured by Meta-Text predicts 12.96% of the outcome variance; and in the best-case scenario, Meta-Text cognition regulation predicts 33.64% of the overall ENEM score.

The result is very interesting because we compared two different measures of the same construct, i.e., the broad metacognitive domain of cognition regulation, one based on self-report and the other based on performance. The self-report measure suggests that this metacognitive domain has no importance in predicting performance on the ENEM, while the performance-based measure suggests the opposite. In short, the results of this study suggest that the performance-based measure is a much better-quality measure of metacognition in the broad domain of cognition regulation. Our criterion, of course, is prediction.

Finally, we verified an important result found in the complete model tested (Table 3). The correlation between Meta-Text cognition regulation and MSLQ cognition regulation was 0.002 (*p* = 0.974), and the correlation between Meta-Text judgment and MSLQ cognition regulation was − 0.007 (*p* = 0.941), i.e., both were null. This result is not new. The results of Castillo-Diaz and Gomes (2023) also showed a null correlation between self-report test measures and performance-based test measures, which were supposedly designed to measure the same construct. These results are worrying and raise doubts about the quality and even the validity of self-report tests in the broad metacognitive domain of cognition regulation.

We may state that self-report tests of cognitive regulation measure and assess metacognitive knowledge rather than cognitive regulation. In our view, metacognitive knowledge can be strongly correlated with cognitive regulation when individuals have very accurate knowledge about the processes involved in cognitive regulation and how this regulation occurs within themselves. When this knowledge is flawed, the correlation may weaken or even become null.

We need to point out that the evidence unfavorable to self-report tests is concentrated on measures of the broad metacognitive domain of cognition regulation. The same does not apply to the broad metacognitive domain referred to in the literature as metacognitive knowledge. This is an important caveat in order to define the scope of our critique.

Among the various impacts of the higher-quality measure is the improved accuracy in analyzing the relationship between the measured construct and convergent and divergent constructs proposed by different models and theories in the field. The evidence from this study has implications for the scientific community investigating metacognition.

Higher-quality measures also have practical impacts on society. For instance, consider a group of psychologists intervening in the cognitive regulation of university students and using a measure with substantial noise to assess cognitive regulation. It is highly likely that the results would underestimate or overestimate the intervention's impact. Furthermore, if a psychologist aims to formulate a diagnosis and uses a cognitive regulation measure with significant noise, the diagnosis is likely to be influenced by that noise, potentially compromising the quality of the diagnosis.

In summary, the results of this study provide evidence that can contribute to both the scientific community in metacognition studies and society. Our study suggests that the agenda of developing and investigating performance-based tests is highly promising. We hope that our article encourages researchers in the field of metacognition to invest in this agenda.

## Conclusion

The results found corroborate the first hypothesis of this study, indicating the superiority of performance-based metacognitive tests in predicting academic performance compared to self-report tests. Prediction generates information that often allows theoretical models to be improved, as well as providing information on possible causal relationships between certain theoretical constructs. In psychometrics, prediction is a very relevant aspect for assessing the quality of tests. For example, a neuropsychological test for the initial detection of dementia is of no importance if it does not predict dementia in its initial state. AERA/APA/NCME (2014) emphasizes the relevance of investigating the predictive validity of tests in educational contexts. Predictive evidence is very important for evaluating the quality of metacognition tests because there is a consensus in the field of metacognitive studies that metacognition is a fundamental construct for improving academic performance. In theory, metacognitive tests that better express this fundamental relationship would be better than tests that do not. This hypothesis is fully corroborated in the results of this study, since there is no correlation between the performance-based metacognitive measure and the self-report-based measure. This result suggests that the two measures of metacognitive regulation are probably not measuring the same construct. Considering that the prediction of the self-report-based test that measures the domain of cognition regulation showed no prediction of academic performance and the performance-based test showed good prediction, it can be assumed that there are problems with the self-report-based tests. The result of this study was the same as the study by Castillo-Diaz and Gomes (2023). Both used different self-report tests and tests that are highly recognized in the metacognitive field.

Our criticism of self-report tests is limited exclusively to tests that measure the broad metacognitive domain of cognition regulation and its components. We believe that self-report is a very appropriate approach for measuring the broad metacognitive domain of metacognitive knowledge and its components.

Considering the similar evidence from our study and that of Castillo-Diaz and Gomes (2023), we highlight the need for caution when making diagnoses and predictions based on self-report-based tests that measure the metacognitive domain of cognition regulation and its components, which is in line with the arguments presented by Craig et al. (2020) that performance-based measurement has little or no association with self-report-based measurement.

Comparing the results of our study with the results of meta-analyses shows that performance-based standardized tests predict academic performance similarly to tasks that require performance. Ohtani and Hisasaka (2018) and Dent and Koenka (2016) show that the prediction of academic performance by metacognitive tasks varies, respectively, between 9.61% and 27.04% and between 11.56% and 18.49% (95% CI). The prediction of performance-based tests found in our study, in the studies by Castillo-Diaz and Gomes (2022, 2023), in the study by Gomes and Golino (2014), and in the study by Gomes et al. (2014), are in this prediction range. This result is very interesting, since these standardized tests are easy to apply and correct, especially the Meta-Text, making it accessible to study metacognitive constructs in large samples. In contrast, cognitive tasks have a complex collection and correction process, restricting the study to small samples.

Regarding the limitations of this study, we would like to point out that our sample consisted only of Brazilian students, predominantly from private institutions and from the humanities and social sciences. In addition, our study did not include in the predictive analysis other variables that influence academic performance, such as intelligence. The study by Castillo-Diaz and Gomes (2022) has already conducted an initial investigation into the predictive role of a performance-based metacognitive test on academic performance when controlling for intelligence. Initial evidence indicated that monitoring, a specific component of cognitive regulation, is as important as intelligence in predicting academic performance. Future studies may include new samples and other variables in the analysis.

## Data Availability

The data that support the findings of this study are available from the corresponding author upon request.

## Abbreviations

MSLQ: Motivated Strategies for Learning Questionnaire
ENEM: Exame Nacional do Ensino Médio
MAI: Metacognitive Awareness Inventory
RC: Regulation of cognition
CFI: Confirmatory Fit Index
TLI: Tucker Lewis Index
GFI: Goodness-of-Fit Index
RMSEA: Root Mean Square Error of Approximation
SRMR: Standardized Root Mean Square Residual
SD: Standard deviation
MIN: Minimum
MAX: Maximum
CI: Confidence interval
*χ*^2^(df): Chi-square (degrees of freedom)
*α*: Cronbach’s alpha
*β*: Standardized beta
*ω*: McDonald’s omega
AERA: American Educational Research Association
APA: American Psychological Association
NCME: National Council on Measurement in Education
